# *Phyllostachys edulis* Compounds Inhibit Palmitic Acid-Induced Monocyte Chemoattractant Protein 1 (MCP-1) Production

**DOI:** 10.1371/journal.pone.0045082

**Published:** 2012-09-18

**Authors:** Jason K. Higa, Zhibin Liang, Philip G. Williams, Jun Panee

**Affiliations:** 1 Department of Cell and Molecular Biology, University of Hawaii John A. Burns School of Medicine, Honolulu, Hawaii, United States of America; 2 Department of Chemistry, University of Hawaii at Manoa, Honolulu, Hawaii, United States of America; University of Leuven, Rega Institute, Belgium

## Abstract

**Background:**

*Phyllostachys edulis* Carriere (Poaceae) is a bamboo species that is part of the traditional Chinese medicine pharmacopoeia. Compounds and extracts from this species have shown potential applications towards several diseases. One of many complications found in obesity and diabetes is the link between elevated circulatory free fatty acids (FFAs) and chronic inflammation. This study aims to present a possible application of *P. edulis* extract in relieving inflammation caused by FFAs. Monocyte chemoattractant protein 1 (MCP-1/CCL2) is a pro-inflammatory cytokine implicated in chronic inflammation. Nuclear factor kappa-light-chain-enhancer of activated B cells (NF-κB) and activator protein 1 (AP-1) are transcription factors activated in response to inflammatory stimuli, and upregulate pro-inflammatory cytokines such as MCP-1. This study examines the effect of *P. edulis* extract on cellular production of MCP-1 and on the NF-κB and AP-1 pathways in response to treatment with palmitic acid (PA), a FFA.

**Methodology/Principal Findings:**

MCP-1 protein was measured by cytometric bead assay. NF-κB and AP-1 nuclear localization was detected by colorimetric DNA-binding ELISA. Relative MCP-1 mRNA was measured by real-time quantitative PCR. Murine cells were treated with PA to induce inflammation. PA increased expression of MCP-1 mRNA and protein, and increased nuclear localization of NF-κB and AP-1. Adding bamboo extract (BEX) inhibited the effects of PA, reduced MCP-1 production, and inhibited nuclear translocation of NF-κB and AP-1 subunits. Compounds isolated from BEX inhibited MCP-1 secretion with different potencies.

**Conclusions/Significance:**

PA induced MCP-1 production in murine adipose, muscle, and liver cells. BEX ameliorated PA-induced production of MCP-1 by inhibiting nuclear translocation of NF-κB and AP-1. Two O-methylated flavones were isolated from BEX with functional effects on MCP-1 production. These results may represent a possible therapeutic application of BEX and its compounds toward alleviating chronic inflammation caused by elevated circulatory FFAs.

## Introduction

Chronic inflammation is associated with obesity and many other diseases of the metabolic syndrome such as type II diabetes, rheumatoid arthritis, and atherosclerosis [1]. Elevated concentrations of free fatty acids (FFAs) in the circulatory system of obese individuals is a common link between chronic inflammation and obesity [2]. FFAs activate pro-inflammatory pathways and induce production of inflammatory cytokines such as MCP-1 [3]. MCP-1 is known to mediate chronic inflammation and contribute to monocyte infiltration into adipose tissue [4], [5]. Accumulation of macrophages in adipose is another hallmark of obesity and other diseases associated with chronic inflammation [6], [7].

Nuclear factor kappa-light-chain-enhancer of activated B cells (NF-κB) and activator protein 1 (AP-1) are transcription factors that activate inflammatory pathways in many cell types [8], [9]. NF-κB is a heterodimeric complex formed from two subunits, of which p50 and p65 (RelA) are the most common [10]. NF-κB is found in all cells and is typically held in an inactive state by being bound to an inhibitor [8]. These inhibitors keep NF-κB sequestered in the cytoplasm, preventing it from activating target genes such as those involved in inflammation [10]. However, the ubiquitous nature of NF-κB allows it to act as a faster mediator of cellular response to inflammatory signals than pathways that require *de novo* synthesis of regulatory proteins [8]. NF-κB is activated by a number of receptors, including Toll-like receptors (TLRs) [11]. FFAs are ligands for TLR2 and TLR4, both of which activate NF-κB in response to FFAs [12].

AP-1 is another dimeric protein complex composed of subunits that bind to a common AP-1 response element [13], [14], and is typically formed from c-Fos and c-Jun [15]. Like NF-κB, AP-1 is also a transcription factor involved in activating inflammatory response genes [16]. c-Fos activity is linked to MCP-1, as suppression of c-Fos inhibits MCP-1 expression [17]. c-Jun is phosphorylated by mitogen-activated protein kinase c-Jun N-terminal kinase (JNK) in response to inflammatory stimuli [18], [19], and is also associated with MCP-1 expression [17]. AP-1 is also linked to NF-κB, as suppressing NF-κB activity prevents AP-1 activation and c-Fos expression [20].

*Phyllostachys edulis* Carriere, also known as “maozhu” in Mandarin or “moso” in Japanese, is a bamboo species used for multiple purposes such as construction, furniture, nutrition, and traditional Chinese medicine [21]. *P. edulis* leaves are currently a byproduct of timber harvesting and production of this species. *P. edulis* grows and proliferates rapidly and is considered an invasive species in areas of China due to its ability to overtake biomes [22]. Potential medical uses of compounds and extracts derived from the leaves of *P. edulis* and other bamboo species have been investigated [23], [24], [25], [26], including possible use towards the treatment of inflammation [27], [28], [29].

Tricin is a flavone first isolated from *Medicago sativa* (alfalfa) [30] and many other sources including rice bran, oats, and bamboo leaves [31], [32], [33]. Tricin has showed anti-tumor [34], [35], [36] and anti-inflammatory properties [37], [38] *in vitro* and *in vivo*. 7-*O*-methyl-tricin (7 MT) has been isolated from other plant sources [31], [39], and has showed antioxidant [40], anti-cancer [40], [41], and cytotoxic properties [42]. Both tricin and 7 MT are O-methylated flavones differing only by methylation of a phenolic hydroxyl group at position 7 (C-7).

Studies on the transcriptional regulation of MCP-1 gene have highlighted stimulus-specific and tissue-specific mechanisms [43], [44]. Recent publications have reported increases of MCP-1 expression in adipose [45] and liver [46] tissues under obese conditions. However, low levels of inflammatory markers were reported in the skeletal muscles in obese subjects [47], and body weight-related changes of these markers in skeletal muscles have not been sufficiently studied.

This study investigates the effects of *P. edulis* bamboo extract (BEX) and two compounds isolated from BEX on MCP-1 overproduction and FFA-activated inflammatory pathways that regulate MCP-1 in cell models of adipose, muscle, and liver cells. We hope this study will further elucidate the effects of BEX and further establish *P. edulis* as a possible therapeutic resource towards alleviating inflammation associated with diseases and complications linked to high systemic free-fatty acids. We were able to show a decrease in MCP-1 expression on the mRNA and protein level, an inhibition of two inflammatory pathways that regulate MCP-1 expression, and identify two functional BEX compounds that inhibit MCP-1 production.

## Results

### *Phyllostachys Edulis* Extract Inhibits MCP-1 mRNA Expression in a Time-dependent Manner

**Figure 1:**
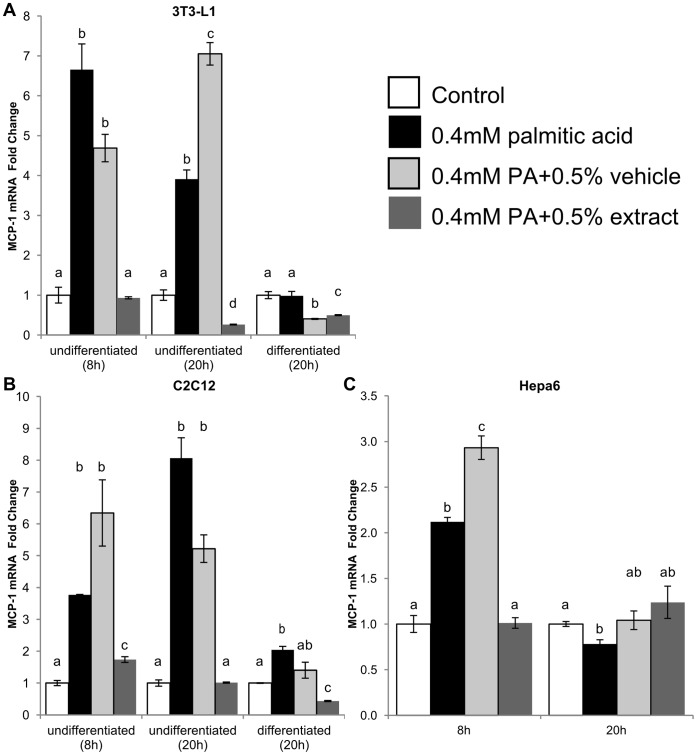
Effect of BEX on MCP-1 mRNA expression. Levels of MCP-1 mRNA expression in 3T3-L1 (A), C2C12 (B), and Hepa6 (C) murine cells in response to palmitic acid (PA, 0.4 mM) in combination with BEX (125 µg/ml or 0.5%, v/v) or ethanol vehicle (0.5%, v/v) at different time points and differentiation states (3T3-L1 and C2C12). Fold change calculated via comparative cycle threshold (−ΔΔCt) normalized to beta-glucuronidase (GUSβ). Other housekeeping genes such as 18 S ribosomal RNA (18 S), hypoxanthine-guanine phosphoribosyltransferase (HPRT), and glyceraldehyde 3-phosphate dehydrogenase (GAPDH) were also used and yielded similar results as GUSβ. Mean ± standard deviation of at least 4 qRT-PCRs per cell type. Mean results were compared between treatments of the same cell type and at the same time point. Differences between means are statistically significant if columns do not share any common letters (p<0.05, one-way ANOVA with Tukey’s multiple comparison test).

PA increased MCP-1 mRNA expression after 8 and 20 hours of treatment in undifferentiated but not differentiated 3T3-L1 cells. Addition of BEX to undifferentiated 3T3-L1 cells reduced MCP-1 expression to a level near that of untreated cells. BEX reduced MCP-1 mRNA expression in differentiated 3T3-L1 cells, but not significantly lower than ethanol vehicle controls (Figure 1A).

MCP-1 mRNA increased in response to PA in both undifferentiated and differentiated C2C12 cells at all time points. In undifferentiated myoblasts, BEX reduced mRNA expression to a similar level as untreated controls, and in differentiated myotubes, BEX lowered expression below that of untreated controls (Figure 1B).

Hepa6 cells showed a similar trend at 8 hours of treatment, as PA increased MCP-1 mRNA and BEX reduced MCP 1-mRNA expression near control levels. However, these differences were not observed later at 20 hours (Figure 1C).

**Figure 2:**
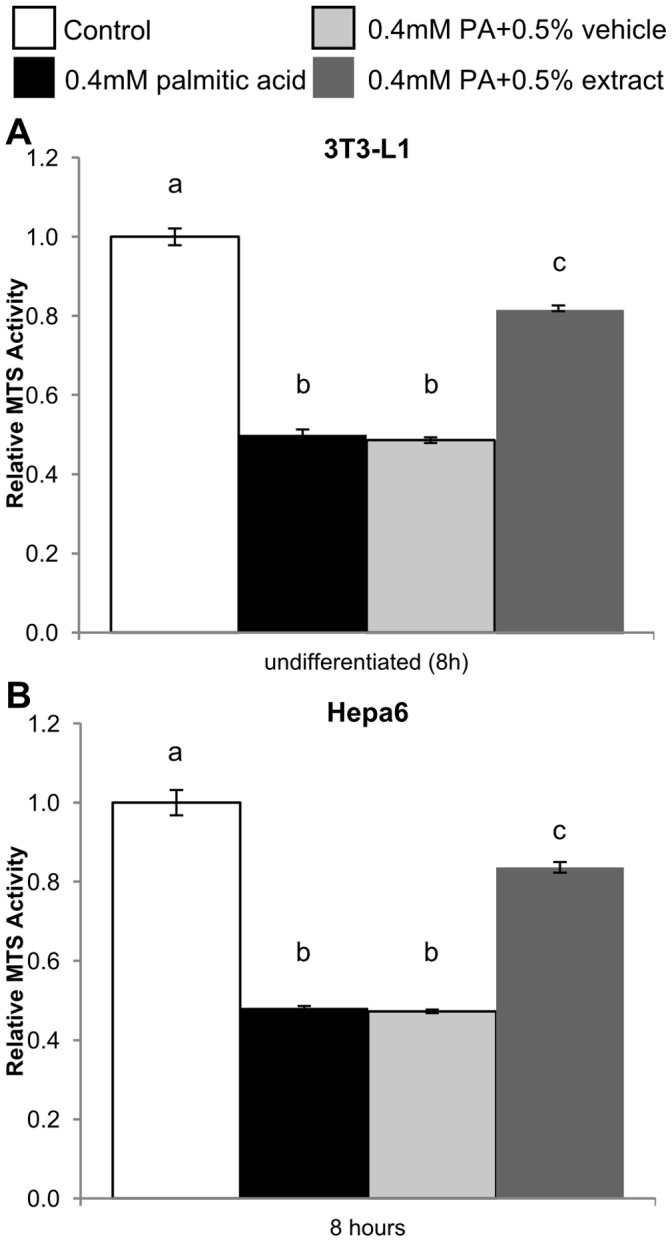
Protective effect of BEX on palmitic acid (PA)-induced lipotoxicity. 3T3-L1 (A), and Hepa6 cells (B) were grown to confluency in 96-well plates and treated with 0.4 mM PA for 24 hours in combination with BEX (125 µg/ml or 0.5%, v/v) or ethanol (0.5%, v/v) as a solvent control. Cell viability was measured using a MTS assay. Mean ± standard deviation over 5 trials, n ≥3 wells per trial. Differences between means are statistically significant if the columns do not share any common letters (p<0.001, one-way ANOVA, Tukey’s multiple comparison test).

To rule out that the reduction in MCP-1 expression was due to cytotoxic effects from BEX, an MTS assay was used to assess whether BEX affected the viability of 3T3-L1 and Hepa6 cells (Figure 2). Exposure of the cells to PA for 24 hours significantly decreased viability of both cell lines, but toxicity from PA exposure was prevented by addition of BEX. Previously, we reported that BEX prevented PA-induced lipotoxicity in C2C12 cells without any harmful effects from BEX alone [48]. The observation that cells die and detach after succumbing to the lipotoxic effects of palmitic acid at around 24 hours may partially explain the dampened response of differentiated and Hepa6 cells to longer exposure to PA, and the results could reflect severely compromised cellular function shortly prior to lipoapoptosis. Taken together, since BEX improves cell viability in the presence of PA, The inhibitory effect of BEX on MCP-1 production is not due to cytotoxicity.

### *Phyllostachys Edulis* Extract Reduces MCP-1 Protein Production from Murine Cell Lines Treated with Palmitic Acid

**Figure 3:**
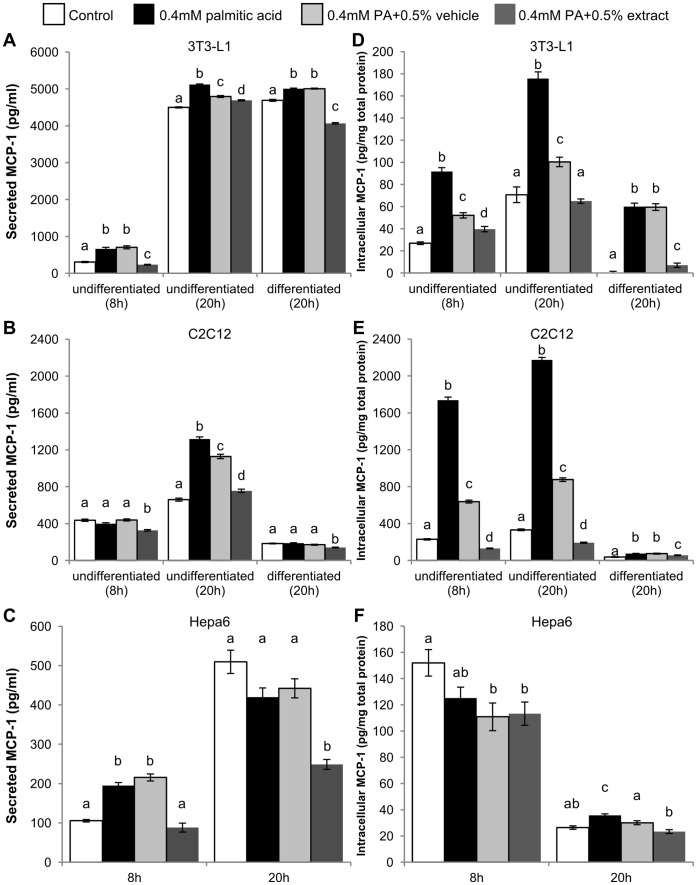
Effect of BEX on lipotoxic MCP-1 production. Production of MCP-1 was measured in the cell media supernatant (A–C) and cytosolic fraction (D–F) of 3T3-L1 (A, D), C2C12 (B, E), and Hepa6 (C, F) murine cells treated with palmitic acid (PA, 0.4 mM) in combination with BEX (125 µg/ml or 0.5%, v/v) or ethanol vehicle (0.5%, v/v). Concentrations were determined by cytometric bead array immunodetection against a reconstituted MCP-1 standard. Mean ± standard error over 3 trials, n ≥300 beads in any trial. Mean results were compared between treatments of the same cell type and at the same time point. Differences between means are statistically significant if columns do not share any common letters (p<0.05, one-way ANOVA with Bonferroni’s post hoc test).

PA increased MCP-1 secretion in both undifferentiated and differentiated 3T3-L1 adipocyte-like fibroblasts. BEX treatment reduced MCP-1 secretion below basal levels in undifferentiated 3T3-L1cells after 8 hours and in differentiated cells after 20 hours. Treatment with an ethanol vehicle control did not induce a similar reduction in MCP-1 as BEX (Figure 3A). C2C12 myoblasts showed a similar increase in MCP-1 secretion in response to PA and reduction with BEX after 20 hours of treatment, but not at 8 hours. Differentiated C2C12 myotubes showed no significant changes in MCP-1 secretion in response to PA and BEX (Figure 3B). PA increased secretion of MCP-1 by Hepa6 cells at 8 hours, but this trend did not continue at 20 hours. BEX reduced Hepa6 MCP-1 secretion below control levels at both time points. (Figure 3C).

Quantitation of intracellular MCP-1 yielded a similar trend in 3T3-L1 cells. PA increased 3T3-L1 intracellular MCP-1 concentrations while BEX decreased MCP-1 in both undifferentiated and differentiated cells, although ethanol also appeared to reduce MCP-1 in undifferentiated cells (Figure 3D). Differentiated C2C12 myotubes contained far less intracellular MCP-1 compared to undifferentiated myoblasts. Similar to 3T3-L1 cells, the addition of ethanol to C2C12 cells reduced intracellular MCP-1, but BEX reduced MCP-1 concentrations even further to a level below vehicle controls (Figure 3E). BEX did not appear to significantly affect intracellular MCP-1 levels in Hepa6 cells (Figure 3F).

### *Phyllostachys Edulis* Extract Inhibits Nuclear Translocation of NF-κB and AP-1 Transcription Factors

3T3-L1 were selected as the primary cell model for further studies of possible mechanisms behind the inhibitory effect of BEX, as 3T3-L1 cells simulate cellular components in adipose which are highly active in obesity-associated chronic inflammation [49].

**Figure 4:**
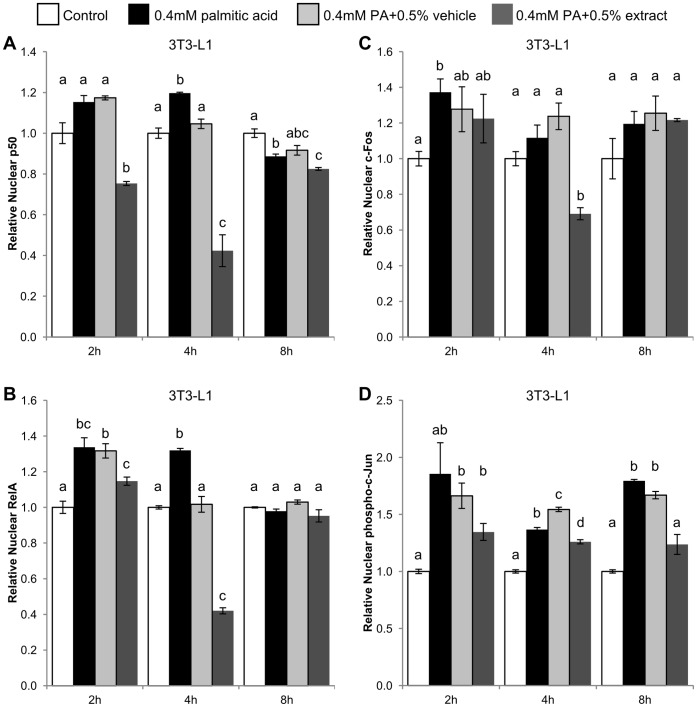
Effect of BEX on nuclear translocation of NF-κB and AP-1 transcription factors over time. Nuclear translocation of NF-κB (A) p50, (B) RelA/p65, AP-1 (C) c-Fos, and (D) phospho-c-Jun subunits in 3T3-L1 cells. Measurements are relative absorbance at 450 nm from a response-element DNA binding colormetric ELISA on nuclear extracts from cells treated with palmitic acid (PA, 0.4 mM) in combination with BEX (125 µg/ml or 0.5%, v/v) or ethanol vehicle (0.5%, v/v). Mean ± standard error over 3 trials, n ≥3 wells per trial. Mean results were compared between treatments at the same time point. Differences between means are statistically significant if columns do not share any common letters (p<0.05, one-way ANOVA with Tukey’s multiple comparison test).

PA increased nuclear concentrations of NF-κB subunits RelA (p65) and p50 in 3T3-L1 cells after 2 hours, although this effect was only significant for RelA (Figures 4A, 4B). At that time, ethanol controls did not affect NF-κB subunit nuclear translocation. BEX inhibited nuclear translocation of both subunits, and for p50, BEX reduced nuclear concentrations below basal levels. After 4 hours of PA treatment, nuclear concentrations of both subunits were comparable to those at 2 hours, although ethanol decreased nuclear concentration of NF-κB subunits to a level near untreated controls. Overall, the effect of BEX on NF-κB was noticed at 2 hours and more evident at 4 hours, as BEX reduced concentrations of both NF-κB subunits to levels far below that of the ethanol vehicle controls at both time points. After 8 hours, the effects of PA and BEX on NF-κB were dampened, with little difference in nuclear concentrations of p50 and RelA between PA, BEX, and controls.

After 2 hours, PA increased nuclear concentrations of the AP 1 subunit c-Fos, but BEX did not show any inhibitory effect at that time (Figure 4C). After 4 hours, however, BEX showed an inhibitory effect and reduced c-Fos to a level below that of other treatments, including untreated controls. After 8 hours, BEX loses its inhibitory effect on c-Fos. Nuclear concentrations of phosphorylated c-Jun increased in response to PA at all time points with a slight exception at 2 hours, in which the increase was not statistically significant. BEX generally lowered levels of nuclear phospho-c-Jun at all time points, and although the reduction was not significant at 2 hours, the effect of BEX was significant at 4 hours and persisted after 8 hours of treatment.

### Flavonoids Isolated from *Phyllostachys Edulis* have Inhibitory Effects on MCP-1 Production

**Figure 5:**
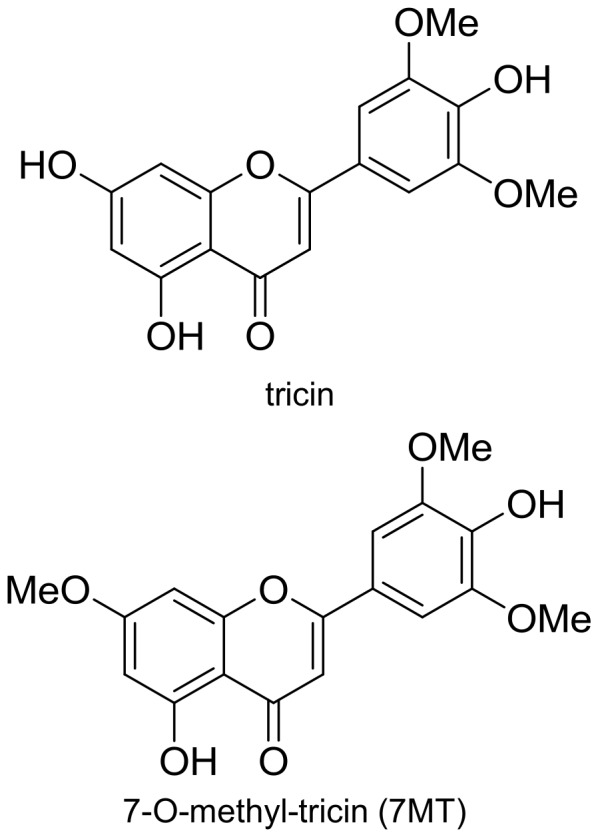
Molecular structures of tricin and 7-O-methyl-tricin (7 MT), two compounds isolated from BEX.

**Figure 6:**
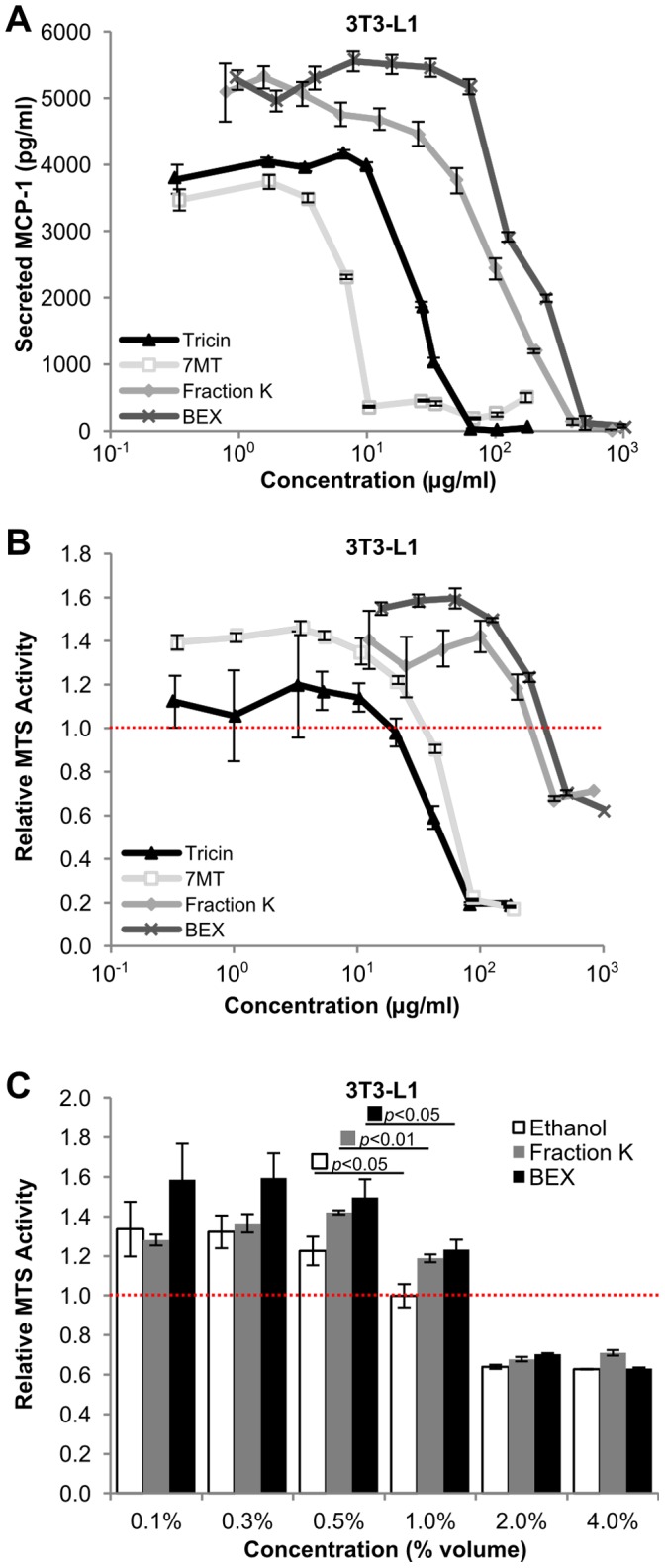
Dose-dependent effects of tricin, 7-O-methyl-tricin (7 MT), Fraction K, and BEX on lipotoxic MCP-1 production and cell viability. (A) MCP-1 concentrations in cell culture media from 3T3-L1 cells cultured in 96 well plates and treated for 20 hours with PA (0.4 mM) and one of the following: tricin (black triangles), 7 MT (light grey hollow squares), Fraction K (grey diamonds), or BEX (dark grey crosses). Concentrations were determined by cytometric bead array immunodetection against a reconstituted MCP-1 standard. Mean ± standard error of 3 samples per dose, n ≥300 beads per sample. (B) Viability of 3T3-L1 cells treated with tricin (black triangles), 7 MT (light grey hollow squares), Fraction K (grey diamonds), or BEX (dark grey crosses) added to maintenance medium. All values are normalized to 3T3-L1 cells from the same plate treated with maintenance medium without flavonoids or PA (normalized average represented by a red dashed line). Mean ± standard error of three trials, n ≥3 per trial. (C) Viability of 3T3-L1 cells treated with ethanol vehicle (outlined white), Fraction K (grey), or BEX (black). Mean ± standard error of three trials, n ≥4 per trial. All values normalized to 3T3-L1 cells from the same plate treated with only maintenance medium (normalized average represented by a red dashed line). *p* values were obtained by performing a Student’s *t*-test between doses.

BEX was fractioned and tested on 3T3-L1 cells with the same PA-induced inflammatory model to determine whether BEX contained specific compounds that inhibited MCP-1 production. Fractions from BEX were tested for bioactivity, and went through several rounds of repeated fractions and assays. Eventually tricin and 7-O-methyl tricin (7 MT) (Figure 5) were isolated from these fractions and found to compose the majority of these fractions. The chemical structure of these natural products was deduced through a combination of NMR experiments, which showed good agreement with reported data [33], [42]. Inhibitory concentrations (ICs) of the compounds were determined by measurement of MCP-1 secreted by confluent 3T3-L1 cells treated for 20 hours with PA in combination with tricin or 7 MT in doses from 0.1 to 500 µM (Figure 6A). The IC50 values are 22.5 µg/ml (68 µM) for tricin and 6.9 µg/ml (20 µM) for 7 MT, suggesting that 7 MT is the more effective than tricin at inhibiting MCP-1 production.

To account for whether the reduction in MCP-1 secretion was due to possible toxicity of the compounds rather than mechanisms that specifically affect MCP-1 production, confluent 3T3-L1 cells were treated for 20 hours with maintenance medium that contained tricin or 7 MT added in doses from 0.1 to 500 µM (Figure 6B). After treatment, cells were rinsed with PBS and an MTS assay was performed to determine viability. The IC50 values on cell viability were 110 µM for tricin and 137 µM for 7 MT.

To compare the effectiveness of the compounds with crude BEX and Fraction K (the BEX fraction from which tricin and 7 MT were isolated, see Materials and Methods), 3T3-L1 cells were again treated with medium that contained PA with BEX (stock concentration of 25 mg/ml in ethanol) or Fraction K (stock concentration of 20 mg/ml in ethanol) in doses up to 1 mg/ml. After incubation for 20 hours, media concentrations of MCP-1 secreted by the cells were measured (Figure 6A). The IC_50_ values were 149 µg/ml for BEX, and 105 µg/ml for Fraction K. To rule out the possibility of cytotoxic effects, an MTS assay was performed on 3T3-L1 cells treated with medium that contained BEX or Fraction K. The inhibitory concentrations for cell viability were roughly double that of the MCP-1 inhibitory concentrations, with IC_50_ values of 290 µg/ml for BEX and 211 µg/ml for Fraction K (Figure 6B). However, doses greater than 125 µg/ml required more than the 0.5% volume used in previous experiments. Between 0.1% and 0.5% (v/v) concentrations, there was no significant change (*p*≥0.10) in cell viability. However, there was a significant drop (*p*≤0.05) in cell viability for ethanol as well as Fraction K and BEX between concentrations of 0.5% and 1.0% (Figure 6C).

## Discussion

Mankind has used plants for medicinal purposes throughout history. One of the earliest recorded examples is the use of salicylates from willow tree bark to alleviate fever since 4th century BCE [50]. Extracts derived from other bamboo species have shown potential use in mediating inflammation [28], [51], and our study shows that P. edulis extract also has anti-inflammatory effects by reducing MCP-1 secretion and inhibiting inflammatory pathways that control MCP-1 production. We have also isolated and identified tricin and 7 MT as two functional compounds in this extract. These findings elucidate possible cellular mechanisms behind the effects of bamboo extract that complements our previous in vivo study in mice that showed the preventative effect of bamboo extract towards increased blood serum MCP-1 caused by a high fat diet [52].

Overall, MCP-1 mRNA expression was highly responsive to lipotoxic challenges in adipose, liver, and skeletal muscle cell lines (Figure 1), with the exception of differentiated 3T3-L1, which showed no increase in mRNA expression after treatment with PA (Figure 1A). Interestingly, the increase in mRNA was poorly reflected on the protein levels (especially secreted MCP-1) in the differentiated skeletal muscle cells (Figures 3B&E). Since skeletal muscle tissue mainly consists of differentiated cells, this result indicates that skeletal muscle may have little contribution to the systemic chronic inflammation under lipotoxic conditions. Furthermore, mRNA levels in undifferentiated 3T3-L1 cells remained high in response to lipotoxic challenges even after drop-offs in NF-κB and AP-1 activity between 4 and 8 hours (Figure 4), which could be partially due to the self-inhibition of NF-κB by its own activation of IκB expression [53]. The high levels of MCP-1 mRNA at later time points could just be the remaining sum of mRNA that was initially transcribed along with mRNA being actively translated and mRNA that hadn’t been targeted for degradation. It may also be possible that other transcription factors or complexes besides NF-κB and AP-1 are sustaining the expression of MCP-1.

The inhibitory effect of BEX on the NF-κB and AP-1 pathways is similar to the findings of other studies on phytochemicals [54], [55]. However, by measuring the activity of these transcription factors over multiple time points, our results indicate that BEX does not chronically suppress these pathways, but rather causes a dynamic response with fluctuating levels of NF-κB and AP-1 inhibition over time. As seen in Figures 4A and 4B, the suppressive effect of BEX on NF-κB subunit activity is noticeable at 2 hours, most potent at 4 hours, and at 8 hours seems to have completely dissipated. The effect of BEX on AP-1 activity seems asynchronous with NF-κB, as c-Fos and phospho-c-Jun show different trends compared to the NF-κB subunits. Like the NF-κB, c-Fos is most inhibited by BEX at 4 hours. However, unlike NF-κB, the effect of BEX on c-Fos is not apparent earlier at 2 hours. The story becomes more complex with phospho-c-Jun, as it not only shows different timing compared to NF-κB, but appears to be out of sync with c-Fos as well. BEX appears to have a longer lasting effect on phospho-c-Jun, as it was the only subunit that continued to show significant inhibition by BEX at 8 hours. Furthermore, BEX seems to maintain a relatively stable level of nuclear phospho-c-Jun, and does not induce a noticeable drop in phospho-c-Jun nuclear localization at 4 hours, something that occurred with the NF-κB and c-Fos subunits. Despite temporal differences in the translocation of these subunits in response to BEX, the end result of this interplay between transcription factors contributes toward an overall inhibition of MCP-1 production. Further studies on compounds isolated from BEX may help to tease apart the complexity of its effects on pro-inflammatory pathways.

The effects of BEX may be due in part to the presence of tricin and 7 MT, which are flavonoids - organic compounds known to have anti-inflammatory properties [56]. Tricin has been isolated from other plant species [33], [37], [57], [58], and showed effectiveness in reducing inflammation as a dietary supplement [36]. 7 MT is known to have antioxidant properties [41], [59], but has not been directly implicated in reducing inflammation. Our results suggest that while 7 MT may share a similar structure and effects with tricin, it could possibly be more effective and less toxic in the prevention of inflammation as it inhibits MCP-1 production at lower doses (IC50 = 20 µM versus 68 µM for tricin) and shows slightly less harmful effects on cell viability (IC50 = 137 µM versus 110 µM for tricin). Since the two compounds differ by only a methyl group, this effect may be due to selectivity toward proteins or receptors that mediate inflammation. Future studies are needed to see whether the difference in the effective concentrations of these two compounds is due to factors such as aqueous solubility, rates of diffusion, or transport into the cell.

Although the concentration at which tricin inhibits MCP-1 (IC_50_ = 68 µM) is lower than the concentration at which it inhibits cell viability (IC_50_ = 110 µM), the overall similarity of the two dose curves (Figures 6A & 6B) makes it possible that the effects of tricin on MCP-1 production could be due to toxicity. However, in the case of 7 MT, not only is less 7 MT needed to inhibit MCP-1 (IC_50_ = 20 µM), but the difference between the concentration at which 7 MT inhibits MCP-1 and the concentration at which it affects viability (IC_50_ = 137 µM) is much greater than tricin, suggesting that 7 MT is more effective at inhibiting MCP-1 production without affecting viability. With regards to Fraction K and BEX, there is little effect on cell viability at concentrations below 0.5% v/v (100 µg/ml Fraction K or 125 µg/ml BEX). However, at concentrations above 0.5% there is a concurrent drop in cell viability with an equal volume of ethanol (Figure 6C), making it difficult to conclude whether Fraction K and BEX are actually cytotoxic at those doses, or if the decrease in viability is due to the high concentration of ethanol vehicle needed to administer those doses.

The concentrations at which tricin and 7 MT inhibit MCP-1 are comparable to other natural products that also inhibit MCP-1 such as: luteolin (effective from ≥10 µM) [54], naringeninchalcone (25 µM) [55], nobiletin and tangeretin (128 µM) [60], p-coumaric acid (25 µM), quercetin (25 µM), and resveratrol (50 µM) [61]. Although those studies also used 3T3-L1 cells as their primary model, it may be worth noting that those studies were performed in different contexts aside from lipotoxicity and FFAs. Furthermore, these studies observed MCP-1 production under different culture conditions, did not use FFAs to induce MCP-1 production, and instead used different methods of inducing inflammation such as treatment with lipopolysaccharides or tumor necrosis factor-alpha. It should also be noted that this study serves as the first report of isolating functional anti-inflammatory compounds from P. edulis.

The IC_50_ of Fraction K with regards to inhibition of MCP-1 production (105 µg/ml) is lower than that of BEX (149 µg/ml), initially giving an impression that it is more potent than BEX. However, Fraction K only composes about 3% of BEX, and although it was the most effective fraction that prevented lipotoxicity, over 23 times of Fraction K is needed to reach its IC_50_ as compared to the amount of Fraction K in the IC_50_ of BEX (3% of 149 µg/ml = 4.47 µg/ml). This suggests that Fraction K may not be the sole source of the inhibitory effects of BEX on MCP-1 production. Furthermore, the IC_50_ values for tricin and 7 MT are 23 µg/ml and 7 µg/ml, whereas these two compounds compose 0.5% and 0.75% of the mass of Fraction K. This indicates a loss of MCP-1 inhibitory efficiency by 42 and 8 folds for tricin and 7 MT, respectively, during the process of isolation from Fraction K. The decrease in MCP-1 inhibitory efficiency along the major steps of the fractionation may be due to: (i) compromised stability of the functional compounds in the matrix; (ii) the existence of other (more potent) active molecules; (iii) synergistic effects between the known and unknown compounds; (iv) loss of material and possible functional compounds during the fractionation process. Further identification of other active compounds of BEX and systematic studies of the interactions among fractions and compounds will be important in establishing production standards if this extract is used as a therapeutic agent or dietary supplement towards the prevention of inflammation.

## Materials and Methods

### Bamboo Extract Source and Preparation

Bamboo extract was provided by Golden Basin LLC (Kailua, HI, United States), and was made from fresh leaves and small branches of Phyllostachys edulis from Hunan Province, China, through a patented ethanol/water extraction procedure (Chinese invention patent, CN1287848A) [62]. Freeze-dried bamboo extract was further processed at a concentration of 100 mg bamboo extract per ml of 100% ethanol. The mixture was incubated at room temperature for 4 hours, and centrifuged at 10,000×g at 4°C for 10 minutes. The ethanol soluble portion (supernatant, 25 mg dry mass/ml ethanol), was the BEX used in the present study.

### Cell Culture

All cells were purchased from American Type Culture Collection (ATCC, Manassas, VA) and maintained in Dulbecco’s Modified Eagle Medium (DMEM, ATCC) supplemented with 10% fetal bovine serum (FBS, Biomeda, Foster City, CA, United States), and 1% penicillin/streptomycin/glutamine (100× PSG, Gibco/Invitrogen, Carlsbad, CA, United States). Cells were incubated in a cell culture incubator at 37°C with an atmosphere of 95% air, 5% carbon dioxide, and 55% humidity. Cells were grown to confluency in 10 cm tissue culture dishes unless intended for use in a dose curve or viability assay, for which they were grown in 96 well plates instead.

### Myotube Differentiation

C2C12 myoblasts were grown to conﬂuency in the aforementioned cell culture conditions (DMEM+10% FBS), and incubated in DMEM supplemented with 2.5% horse serum (Sigma, St. Louis, MA, United States) for up to 10 days to allow for full differentiation into myotubes [48].

### Adipocyte Differentiation

3T3-L1 pre-adipocytes were grown to conﬂuency and maintained for 2 days, and then treated with 0.5 mM 3-isobutyl-1-methylxanthine(IBMX, Sigma), 10 µg/ml insulin (Sigma, Catalog No. I0516), and 1 µM dexamethasone (Sigma) for 2 days, followed by treatment with 10 µg/ml insulin for 6 days.

### Free-fatty Acid Lipotoxic Induction of Inflammation and BEX Treatment

Palmitic acid (PA, Sigma) was dissolved in dimethylsulfoxide (DMSO, Sigma) at a concentration of 0.4 M. This stock was added to complete culture medium at a 1∶1000 ratio to reach a final concentration of 0.4 mM at 42°C [48]. Cells were treated with: (i) complete medium; (ii) complete medium with 0.4 mM PA; (iii) complete medium with 0.4 mM PA and 125 µg/ml (0.5% v/v) BEX; and (iv) complete medium with 0.4 mM PA and 0.5% (v/v) ethanol as a solvent control. 125 µg/ml BEX was established as a standard dose based on our previous study, which used 0.5% v/v BEX [48].

### mRNA Quantification

RNA was extracted from cells post-treatment using a RNeasy Mini Kit (Qiagen, Valencia, CA, United States). cDNA was synthesized using the SuperScript III First-Strand Synthesis System (Invitrogen). Primer sequences are (from 5′ to 3′): GUSβ forward: CTCTGGTGGCCTTACCTGAT; GUSβ reverse: CAGTTGTTGTCACCTTCACCTC; MCP-1 forward: CATCCACGTGTTGGCTCA; MCP-1 reverse: GATCATCTTGCTGGTGAATGAGT. qRT-PCR was performed in quadruplicate using Platinum SYBR Green qPCRSuperMix-UDG Kit (Invitrogen) (5 µL each reaction) in 384-well plates and quantified using a Light cycler 480 II real-time PCR machine (Roche Applied Sciences, IN, United States).

### Fractionation of Cell Lysate

Cytosolic and nuclear cellular fractions were prepared using a Nuclear Extract Kit (Active Motif, Carlsbad, CA, United States). Protein concentrations of each fraction were measured using a Bradford assay (Bio-rad, Hercules, CA, United States).

### MCP-1 Protein Detection and Quantification

MCP-1 protein was detected in cytosolic fractions and culture media using a Cytometric Bead Array (CBA) – Mouse MCP-1 Flex Set with included MCP-1 lyophilized standard (BD Biosciences, San Jose, CA, United States) and analyzed with a BD FACSCalibur ﬂow cytometer (BD Biosciences). Cytosolic fractions were normalized to 1 mg/ml total protein using a Bradford assay to quantify and phosphate buffered saline to dilute samples.

### Quantification of Transcription Factors

TransAM NF-κB Family Kit and TransAM AP-1 Family Kit (Active Motif) were used to measure concentrations of p50, RelA (p65), c-Fos, and phospho-c-Jun in cellular nuclear fractions via colorimetric DNA-binding ELISA to response element consensus sequences. ELISA readouts were performed on a SpectraMAX 340 (Molecular Devices, Sunnyvale, CA, United States) by measuring absorbance of samples at a wavelength of 450 nm. Nuclear fractions were normalized to 1 mg/ml total protein using assay binding buffer to dilute samples.

### Cell Viability Detection

Cell viability was detected by CellTiter 96® AQueous Non-Radioactive Cell Proliferation Assay (Promega, Madison, WI, United States). Conversion of MTS to formazan by viable cells was quantified by measuring absorbance of samples at a wavelength of 490 nm with a SpectraMAX 340 (Molecular Devices).

### Isolation and Purification of Tricin and 7 MT from *Phyllostachys Edulis*

A powder of *P. edulis* leaves (73 g) was extracted five times with 500 mL of methanol overnight at room temperature to afford a total of 12.8 g of crude extract. A 1.2 g portion of this residue was separated on a DPA-6S polyamide column (500 mg; Supelco Discovery) by vacuum chromatography using a step gradient of 0 (× 3), 25 (× 3), 50 (× 3), 75 (× 3), 100% (× 5) methanol (MeOH) in H_2_O, providing 17 fractions (A–Q) of 729.0, 55.6, 12.3, 15.0, 16.2, 9.6, 21.9, 24.7, 11.6, 26.1, 36.0, 18.1, 41.0, 11.6, 3.1, 1.5, and 0.7 mg, respectively. MTS assay results indicated that the active components were concentrated in fraction K (36.0 mg). LC-MS analyses revealed that fraction K contained a series of compounds with molecular weights between 300 and 350 amu with UV maxima at 270 and 350 nm. Separation of fraction K (36 mg) by reversed-phase HPLC [Luna C8, 250 × 10 mm, a linear gradient over 45 min from 20 to 100% acetonitrile in H_2_O with 0.1% formic acid added to both solvents, flow rate 3 mL/min, PDA detection] afforded 21 subfractions, whose anti-lipotoxic activities were concentrated in subfraction-7 (0.2 mg) and -11 (0.3 mg), respectively. Further purification of these two subfractions using the same conditions as reported above led to the isolation of two known flavones tricin (*t*_R_ 18 min, 0.18 mg, 0.015% yield) and 7 MT (*t*_R_ 24.5 min, 0.27 mg, 0.023% yield).

Tricin: Light yellow powder; ^1^H NMR (500 MHz, DMSO-*d*_6_) δ_H_ 3.87 (s, 6H, 3′-OMe, 5′-OMe), 6.10 (d, *J* = 1.43 Hz, 1H, H-6), 6.44 (d, *J* = 1.43 Hz, 1H, H-8), 6.88 (s, 1H, H-3), 7.28 (s, 2H, H-2′, H-6′); HRESI-TOFMS *m/z* [M+H]^+^331.0813 [calculated for C_17_H_15_O_7_^+^, 331.0818, -1.5 ppm error].

7-Methoxytricin: Light yellow powder; ^1^H NMR (500 MHz, CDCl_3_) δ_H_ 3.89 (s, 3H, 7-OMe), 4.00 (s, 6H, 3′-OMe, 5′-OMe), 6.38 (d, *J* = 2.2 Hz, 1H, H-6), 6.51 (d, *J* = 2.2 Hz, 1H, H-8), 6.58 (s, 1H, H-3), 7.12 (s, 2H, H-2′, H-6′); HRESI-TOFMS *m/z* [M+H]^+^345.0970 [calculated for C_17_H_15_O_7_^+^, 345.0974, -1.2 ppm error].

### Statistical Analyses

Software used was Prism 4.0a (GraphPad Software, Inc., La Jolla, CA, United States). Methods employed in the data analyses included one-way ANOVA with Tukey’s multiple comparison test, ANOVA with post-hoc test and Bonferroni correction, and Student t-test. *p*<0.05 was considered statistically significant.
